# The first complete chloroplast genome of *Fagopyrum leptopodum* (Diels) Hedberg (Caryophyllales: Polygonaceae) with phylogenetic implications

**DOI:** 10.1080/23802359.2021.1945967

**Published:** 2021-07-05

**Authors:** Xueling Ye, Luo Wang, Dabing Xiang, Yanxia Sun

**Affiliations:** Key Laboratory of Coarse Cereal Processing, Ministry of Agriculture and Rural Affairs, Sichuan Engineering & Technology Research Center of Coarse Cereal Industralization, School of Food and Biological Engineering, Chengdu University, Chengdu, P. R. China

**Keywords:** *Fagopyrum*, chloroplast genome, phylogenetic analysis, molecular marker

## Abstract

In the present study, we sequenced and assembled the complete chloroplast genome of *Fagopyrum leptopodum* (Diels) Hedberg. The chloroplast genome of *F. leptopodum* was composed of 85 protein-coding genes, 8 ribosomal RNA genes, and 37 transfer RNA genes. The *F. leptopodum* chloroplast genome is 159,375 bp in length, with a GC content of 37.81%. Phylogenetic analysis based on the combined chloroplast gene dataset indicated that the *F. leptopodum* exhibited a close relationship with *Fagopyrum luojishanense*.

The genus *Fagopyrum* is a gluten-free pseudocereal that belongs to the Polygonaceae family. Some *Fagopyrum* species are highly nutritional food components, which show a variety of nutritional and medicinal value, including anti-inflammatory, plasma cholesterol level reduction, antioxidant, anticancer, neuroprotection, and antidiabetic effects (Ohsawa et al. 2020; Song et al. 2020; Zhou et al. 2020). Because of the high nutritional value of *Fagopyrum* species, scholars pay more and more attention to the cultivation and nutrition of *Fagopyrum* species (Song et al. 2016; Xiang et al. 2016; Xiang, Ma, et al. 2019; Xiang, Song, et al. 2019; Xiang et al. 2020). The genus *Fagopyrum* comprises nearly 30 species, mostly endemic to southern China (Ohsako and Li 2020). The genus *Fagopyrum* has high genetic diversity and contains rich genetic resources. Accurate classification and phylogenetic analysis of the genus *Fagopyrum* will promote the genetic breeding of *Fagopyrum* species. Organelle genomes have been widely used in the study of taxonomy, evolution, and genetics (Li et al. 2019; Yang et al. 2019; Li, He, et al. 2020; Li et al. 2021). However, no complete chloroplast genome of *Fagopyrum leptopodum* was reported to date.

The specimen (*F. leptopodum*) sequenced in the present study was collected from Sichuan, China (101.31E; 27.56 N). A specimen was deposited at the Collection Center of Chengdu University (Dabing Xiang, xiangdabing@cdu.edu.cn) under the voucher number XYQ_B1. We assembled the *F. leptopodum* chloroplast genome according to previously described methods (Li et al. 2021). First, the total genomic DNA of *F. leptopodum* was extracted using a Plant DNA Kit (D3485-00, Omega Bio-Tek, Norcross, GA, USA). And then we purified the extracted genomic DNA using a Gel Extraction Kit (Omega Bio-Tek, Norcross, GA, USA). The purified DNA was stored in Chengdu University (No. DNA_ XYQ_B1). We constructed sequencing libraries of *F. leptopodum* using a NEBNext^®^ Ultra™ II DNA Library Prep Kit (NEB, Beijing, China). A-tailed ligated to paired-end adaptors, and PCR amplified with a 350 bp insert was used for the library construction. Whole genomic sequencing (WGS) of *F. leptopodum* was conducted using the Illumina HiSeq 2500 Platform (Illumina, San Diego, CA). The chloroplast genome of *F. leptopodum* was *de novo* assembled using NOVOPlasty v4.3 (Dierckxsens et al. 2017). We annotated the complete chloroplast genome of *F. leptopodum* using GeSeq (Tillich et al. 2017). The chloroplast genome of *Fagopyrum luojishanense* J. R. Shao (Wang et al. 2017) was set as the reference genome for chloroplast genome assembly and annotation of *F. leptopodum*.

The complete chloroplast genome of *F. leptopodum* is 159,375 bp in length. The base compositions of the *F. leptopodum* chloroplast genome were as follows: A (30.93%), T (31.26%), G (18.59%), and C (19.22%). The complete chloroplast genome of *F. leptopodum* contains 85 protein-coding genes, 8 ribosomal RNA genes, and 37 transfer RNA genes. The *F. leptopodum* chloroplast genomes include a pair of IR regions of 30,848 bp. It was separated by a large single-copy (LSC) region of 84,454 bp and a small single-copy (SSC) region of 13,226 bp. To investigate the phylogenetic status of the chloroplast genome of *F. leptopodum*, we constructed a phylogenetic tree for 21 species. The protein-coding region of 63 genes conserved in the 21 species was used to construct combined a chloroplast gene set (Wang, Wang, et al. 2020; Wu et al. 2021). We used the Bayesian (BI) analysis method to construct the phylogenetic tree based on combined protein-coding genes of the chloroplast genome as described by previous methods (Li, Yang, et al. 2020; Cheng et al. 2021). First, we aligned individual protein-coding genes of chloroplast genomes using MAFFT v7.037 (Katoh et al. 2019) and then concatenated these alignments into a combined gene dataset using SequenceMatrix v1.7.8 (Vaidya et al. 2011). Potential phylogenetic conflicts between different genes were detected by a partition homogeneity test (Wang, Song, et al. 2020); PartitionFinder 2.1.1 (Lanfear et al. 2017) was used to determine best-fit models of evolution and partitioning schemes. MrBayes v3.2.6 (Ronquist et al. 2012) was used to perform the BI analysis. Two independent runs with four chains (three heated and one cold) each were conducted simultaneously for 2 × 10^6^ generations. Each run was sampled every 100 generations. We assumed that stationarity had been reached when the estimated sample size (ESS) was greater than 100 and the potential scale reduction factor (PSRF) approached 1.0. The first 25% of samples were discarded as burn-in, and the remaining trees were used to calculate Bayesian posterior probabilities (BPP) in a 50% majority-rule consensus tree (Ye et al. 2020; Li, He, et al. 2020; Li, Ren et al. 2020). The 5 *Fagopyrum* species could be divided into two groups (Figure 1), wherein the first could be recovered as (*F. esculentum* + (*F. tataricum* + *F. dibotrys*)), and the second group comprised two species, *F. leptopodum* and *F. luojishanense*. According to the phylogenetic tree, the *F. leptopodum* is a sister species to *F. luojishanense* (Wang et al. 2017).

**Figure 1.**
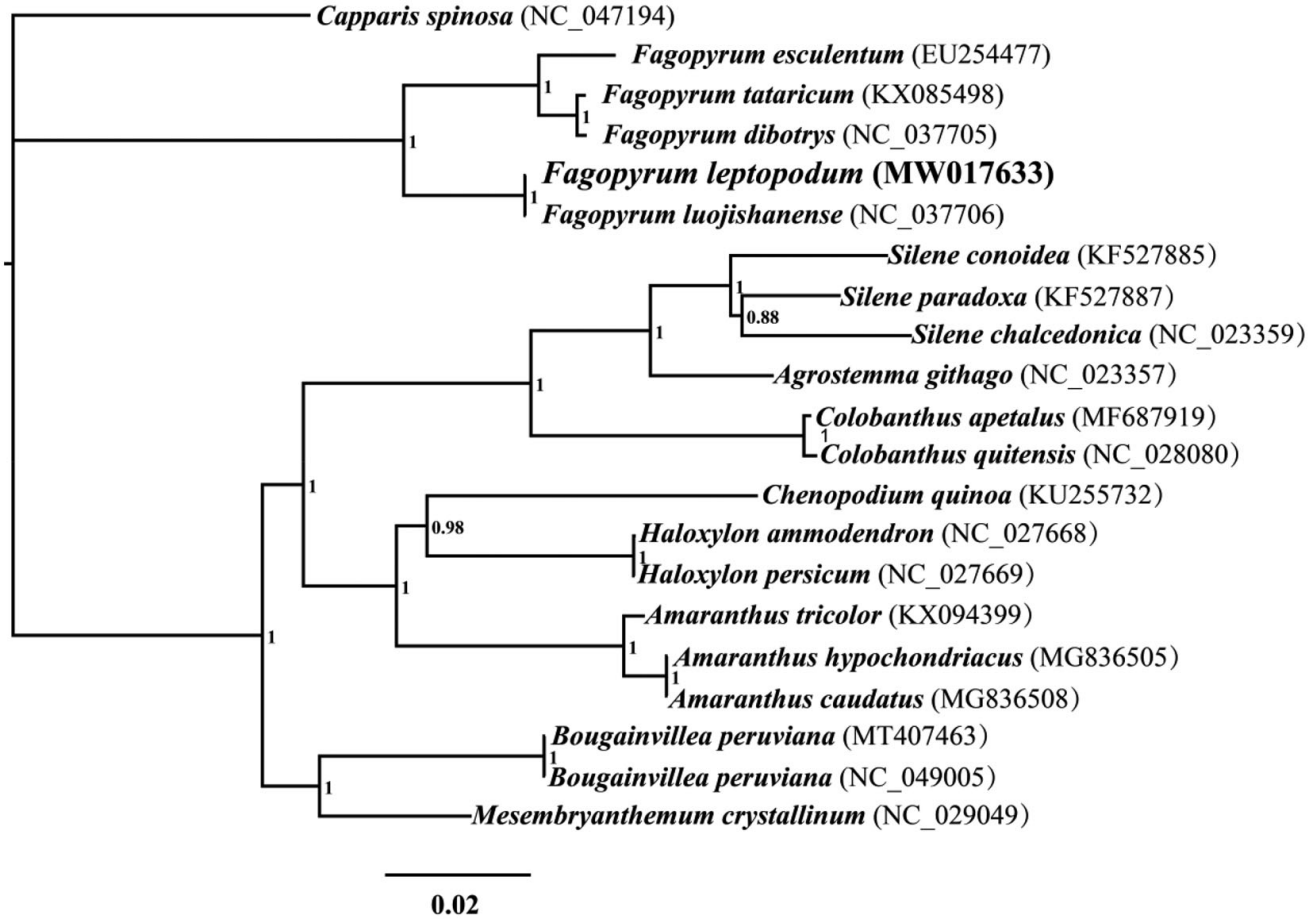
Bayesian phylogenetic analysis of 21 species based on the combined protein-coding genes of chloroplast genome. Support values are Bayesian posterior probabilities (BPP). Accession numbers of chloroplast sequences used in the phylogenetic analysis are listed in brackets after species.

## Data Availability

The genome sequence data that support the findings of this study are openly available in GenBank of NCBI at (https://www.ncbi.nlm.nih.gov/) under the accession no. MW017633. The associated BioProject, SRA, and Bio-Sample numbers are PRJNA717122, SRR14066945, and SAMN18478794, respectively.
